# The potential of *Leishmania donovani* Aurora kinase in the diagnosis of Indian and Brazilian visceral leishmaniasis patients

**DOI:** 10.1128/spectrum.03247-24

**Published:** 2025-04-16

**Authors:** Anirban Bhattacharyya, Rudra Chhajer, Sarfaraz Ahmad Ejazi, Mohd Kamran, Saswati Gayen, Mehebubar Rahman, Rama Prosad Goswami, Krishna Pandey, Vidya Nand Ravi Das, Pradeep Das, Fernando Oliveira da Silva, Dorcas Lamounier Costa, Carlos Henrique Nery Costa, Nahid Ali

**Affiliations:** 1 Infectious Diseases and Immunology Division, Council of Scientific and Industrial Research (CSIR)-Indian Institute of Chemical Biology 30156, Kolkata, West Bengal, India; 2 ICMR-Rajendra Memorial Research Institute of Medical Sciences (RMRIMS) 83429, Patna, India; 3 Department of Microbiology, Vijaygarh Jyotish Ray College, Kolkata, West Bengal, India; 4 Department of Tropical Medicine, School of Tropical Medicine, Kolkata, West Bengal, India; 5 Universidade Federal do Piaui, Teresina, Brazil; Inflammatix Inc, Sunnyvale, California, USA

**Keywords:** Aurora kinase, visceral leishmaniasis, post kala-azar dermal leishmaniasis, diagnosis, ELISA, LFT

## Abstract

Visceral leishmaniasis (VL), caused by *Leishmania donovani* or *Leishmania infantum*, is prevalent in India and Brazil. Post-kala-azar dermal leishmaniasis (PKDL), a cutaneous form, can occur in patients who seem to have recovered from VL. The rK39 test, which detects circulating antibodies, shows high sensitivity and specificity for VL diagnosis in India, but its performance varies in other endemic regions, with a significant limitation being the inability to distinguish active disease from past infection. Herein, we investigated Aurora Kinase (LdAIRK), a conserved virulence factor across *Leishmania* species with a major role in cell division, for VL diagnosis. We analyzed serum samples from parasitologically confirmed symptomatic VL patients (*n* = 79), PKDL patients (*n* = 16), healthy controls, and other diseases (*n* = 53) from India, along with VL patients (*n* = 40) and healthy endemic controls (*n* = 19) from Brazil, using enzyme-linked immunosorbent assay with rLdAIRK. The sensitivity of rLdAIRK was 98.73% (95% CI: 93.17–99.94) for Indian patients and 97.5% (86.84–99.94) for Brazilian patients. It also demonstrated a sensitivity of 93.75% (71.67–99.68) for Indian PKDL sera. Specificity ranged from 94.33% to 94.74% for Indian samples and 84.21% for Brazilian samples. Notably, LdAIRK showed low reactivity (8.3%) with follow-up patient samples compared with rK39 rapid diagnostic tests, indicating its potential as a test-of-cure tool. These findings were supported by dipstick and lateral flow tests (LFTs), which are user-friendly and suitable for field settings. We recommend incorporating recombinant antigen Ldairk in serological assays for the diagnosis of VL.

**IMPORTANCE:**

An early detection and treatment of VL is essential in the control of this potentially fatal disease. Since signs and clinical symptoms of VL are non-specific, diagnosis is confirmed with serological tests. Rapid diagnostic tests (RDTs) against VL that detect antibodies are simple and field adaptable. rK39 antigen-based RDTs are in use, but their sensitivities vary in the different VL-endemic regions. Moreover, since the antibodies persist long after cure in healthy individuals, these RDTs cannot diagnose relapse of the disease. Here, we have identified a Ldairk as a new marker for the diagnosis of VL. We found that Indian VL and PKDL as well as Brazilian patient sera reacted to this protein consistently. Sensitivity was also maintained in the patient’s urine samples. Low reactivity with antibodies after cure with Ldairk can help distinguish previously treated cases from active and relapsed ones.

## INTRODUCTION

Kinetoplastid parasites, responsible for Chagas disease, African trypanosomiasis, and leishmaniasis, are major neglected tropical diseases (NTDs) identified by the World Health Organization (WHO). These diseases are caused by *Trypanosoma cruzi, Trypanosoma brucei*, and over 20 species of *Leishmania* (1). Visceral leishmaniasis (VL), or kala-azar, is one of the most lethal parasitic diseases, with high incidence and mortality rates (2). In India, it is caused by *Leishmania donovani*, whereas in Brazil, *Leishmania infantum* is the primary cause (3). According to the WHO 2021 report, the major VL hotspots are East Africa (66% of global cases), the Indian subcontinent (12%), and Brazil (16%) (https://www.who.int/publications/i/item/9789240010352).

VL initially presents with symptoms similar to other diseases like malaria and dengue, including irregular fever, weight loss, splenomegaly, hepatomegaly, and anemia (4). Laboratory diagnosis is typically done through microscopic examination of Giemsa-stained tissue aspirates, but these methods can be slow and require invasive procedures like bone marrow or splenic aspirations, which are often impractical in field settings (5). The Infectious Diseases Society of America (IDSA) advises against splenic aspiration for VL diagnosis due to its high risk (6). Other diagnostic methods, such as direct agglutination tests (DAT), indirect immunofluorescence (IIF), indirect hemagglutination (IHA), and enzyme-linked immunosorbent assay (ELISA), have limitations, including variable performance and the need for multiple samples (7). Molecular techniques like quantitative real-time PCR (qPCR) and loop-mediated isothermal amplification (LAMP) offer high specificity but require expensive lab equipment (8). Rapid diagnostic tests (RDTs) such as lateral flow assays (LFA), particle agglutination, and immunofiltration are being developed, with the rK39-based assay showing promise in the Indian Subcontinent (9). However, its performance is inconsistent in East Africa and Brazil due to genetic variability in *L. donovani* strains and cross-reactivity with other diseases (10). This highlights the need for new diagnostic antigens to improve VL diagnosis. Our previous work on *Leishmania* membrane antigens (LAg) from *L. donovani* demonstrated strong sensitivity and specificity in detecting infection-specific antibodies, with LAg-based RDTs showing promise as point-of-care tools (11). However, batch-to-batch variability and high production costs of purified antigens remain significant challenges (12). Thus, identifying more refined antigenic biomarkers is crucial to enhancing the sensitivity, specificity, and accessibility of VL diagnostics, including potential tests for cure.

Recent advances in bioinformatics and biotechnology have highlighted protein kinases (PKs) as promising drug targets (13). The leishmanial kinome, comprising 175–195 PKs depending on the species, regulates critical cellular functions such as cell cycle progression, differentiation, and virulence (14). We previously identified a putative Aurora-like kinase in *L. donovani* (LdAIRK), a member of the mitotic serine/threonine kinase family, which plays a key role in cell division (15). LdAIRK’s dynamic localization from the cytoplasm to the nuclear periphery and spindle poles during mitosis emphasizes its importance in orchestrating the cell division cycle, making it a potential therapeutic target (13). Given its essential role in the survival of both promastigotes and amastigotes and its conservation across *Leishmania* species associated with visceral disease, we investigated the immunoreactivity of LdAIRK against antibodies in kala-azar patients. Sera from Indian VL, PKDL, and Brazilian VL patients were screened to assess their potential as a diagnostic marker for VL and validate their ability to differentiate active infections from past exposure.

## RESULTS

### Identification, cloning, and purification of LdAIRK

**Fig 1:**
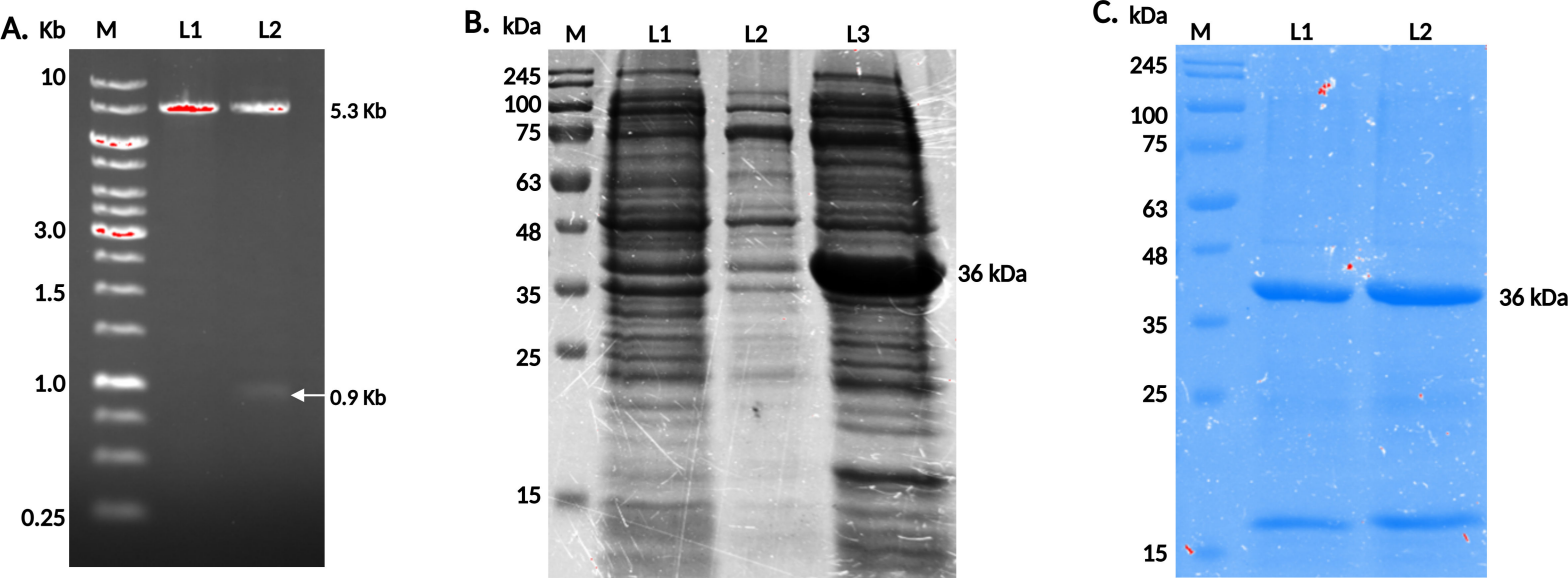
Cloning and expression of recombinant LdAIRK. (**A**) LdAIRK was PCR amplified and cloned into pET28a. Lane M shows base pair markers, with lanes L1 and L2 showing the 5.3 kb pET28a and 0.9 kb LdAIRK bands, respectively. (**B**) LdAIRK expression was induced with 0.3 mM IPTG. Lane M shows molecular markers, lanes 1 and 2 show pre-induction lysates, and lane 3 shows the induced 36 kDa protein. (**C**) Purification of Ldairk. Lane M shows molecular markers, and lanes 1 and 2 display purified recombinant proteins.

LdAIRK (LdBPK_280550.1 AIRK) has 906 base pairs, which encode a protein with 301 amino acids having a molecular weight of about 36 kDa (15). PCR amplification by LdAIRK gene-specific primers was carried out using the genomic DNA of *L. donovani* strain AG83. The amplified product was then cloned. A band of 0.9 kb corresponding to a gene size of LdAIRK in the fall-out experiment is shown (Fig. 1A), which signifies that the gene was cloned successfully. The protein was then expressed by induction with 0.3 mM IPTG in Rosetta (DE3) strain of *E. coli* (Fig. 1B). The expression of Ldairk was found to be maximal in lane 3 in comparison to uninduced lysate in lanes 1 and 2. Furthermore, the confirmed purified recombinant protein by SDS-PAGE is shown in (Fig. 1C). The protein concentration corresponding to the molecular weight of Ldairk was found to be 36 kDa in different elution fractions in lanes 1 and 2.

### Immunoblot assay of Ldairk with serum samples

**Fig 2:**
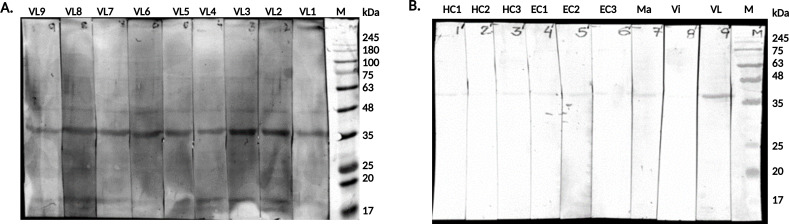
Immunoblot assays against recombinant Ldairk. (**A**) Immunoblot analysis of recombinant Ldairk using serum samples from nine visceral leishmaniasis patients (VL1-VL9). Protein markers are shown in lane 1. (**B**) Immunoblot analysis of Ldairk with various samples: non-endemic healthy controls (HC1-3), endemic healthy controls (EC1-3), other diseases (malaria in lane 7, viral fever in lane 8), and a VL-positive sample (lane 9). Protein markers are in lane M.

The reactivity of the recombinant antigen in the immunoblot assay was assessed using serum samples from VL-positive patients and healthy controls. Ldairk’s interaction with various samples was evaluated in two distinct immunoblot assays. In Fig. 2A, the assay was conducted with serum samples from nine active VL patients (VL1–9). For the control immunoblot assay (Fig. 2B), six samples were employed: three non-endemic healthy controls (HC1, HC2, and HC3), three endemic healthy controls (EC1, EC2, and EC3), two samples from diseases similar to VL, including malaria (Ma) and viral fever (Vi), and one serum sample from an active VL patient (VL1) as a positive control. Ldairk showed consistent positive reactivity with all serum samples from VL patients, appearing at a position of 36 kDa (Fig. 2A). In Fig. 2B, the Ldairk antigen showed negative binding with endemic and non-endemic healthy individuals, as well as other diseases, except for positive binding with the VL sample in lane 9.

### ELISA assay with Ldairk for determining antigen-specific antibodies using Indian and Brazilian serum samples

**Fig 3:**
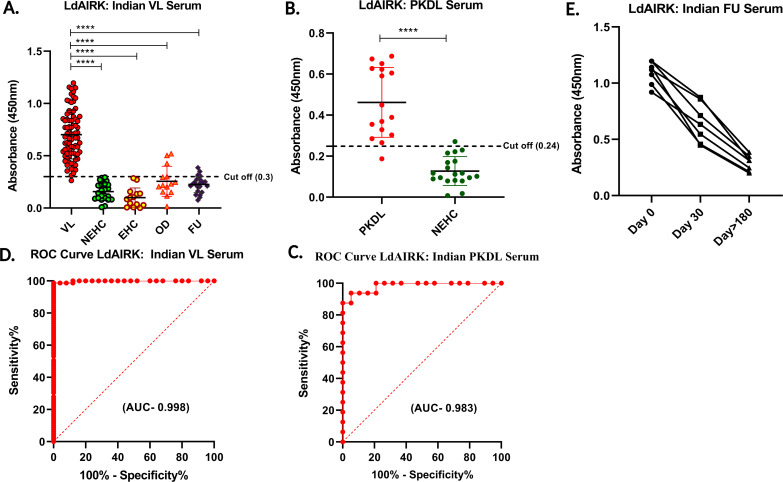
Indian serum-based ELISA with recombinant Ldairk. (**A**) Serum samples from visceral leishmaniasis (VL, *n* = 79), non-endemic healthy controls (NEHC, *n* = 33), endemic healthy controls (EHC, *n* = 14), patients with other diseases (OD, *n* = 14), and follow-up patients (FU, *n* = 24). (**B**) Serum samples from post-kala-azar dermal leishmaniasis (PKDL, *n* = 16) and non-endemic healthy controls (NEHC, *n* = 19). Dotted horizontal lines represent cutoff values (0.29, 0.26) determined by ROC curves (****, *P* < 0.0001). (**C**) (**D**) ROC curves for detecting antigen-specific antibodies in serum using Ldairk. (**E**) Comparison of antibody levels in VL patients' serum (1:2,000 dilution) before treatment (day 0), 1 month after treatment (day 30), and 6 months after treatment (day 180). Paired *t*-tests were used for comparison.

**Fig 4:**
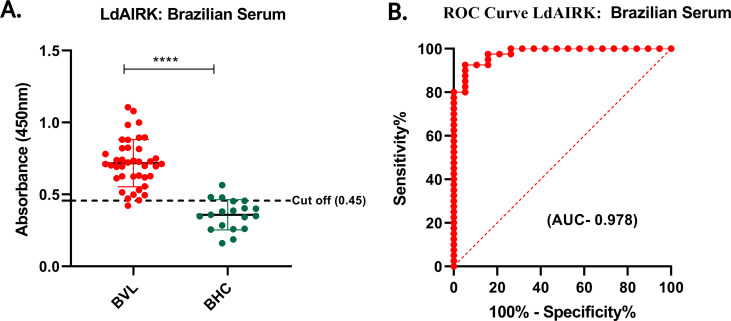
Brazilian serum-based ELISA. (**A**) Serum samples from visceral leishmaniasis patients (BVL, *n* = 40) and healthy controls (BHC, *n* = 19). The dotted horizontal line represents the cutoff value (0.45) determined by ROC curves for optimal sensitivity and specificity (****, *P* < 0.0001). (**B**) ROC curves generated from ELISA assays using LdAIRK antigen to detect antigen-specific antibodies in serum samples.

Following the confirmation of Ldairk’s immunogenicity in the immunoblot assay, we evaluated the diagnostic potential of recombinant Ldairk antigen using ELISA, analyzing serum samples from various groups. These included 79 VL patients, 16 PKDL patients, 85 controls (14 EHC, 33 NEHC, and 14 OD patients) from India, and 40 VL patients and 19 healthy controls from Brazil. Antigen-specific IgG antibodies were measured by optical density at 450 nm. Cutoff values were determined as 0.3 for Indian VL, 0.24 for PKDL, and 0.45 for Brazilian VL sera. The sensitivity of 98.73% (95% CI: 93.17–99.94) for Indian VL, 93.75% (95% CI: 71.67–99.68) for PKDL, and 97.5% (95% CI: 86.84–99.94) for Brazilian VL confirm the assay’s effectiveness in detecting positive cases of VL for each respective type. Specificity of 94.33% (95% CI: 84.63–98.46), 94.74% (95% CI: 75.36–99.73), and 84.21% (95% CI: 60.42–96.62), respectively, were observed for healthy controls for these three studies (Fig. 3A and B, Fig. 4A, Table 1). Additionally, the area under the curve (AUC) for total IgG of the Ldairk antigen with VL samples was 0.987 close to that observed with PKDL 0.983 and Brazilian serum samples with 0.978. Furthermore, antibody levels of VL patients in India were statistically significant compared with endemic healthy controls and other diseases. ROC curves obtained from Indian VL, PKDL, against NEHC, and Brazilian sera against HC for the antigen Ldairk are shown in Fig. 3C and D, and Fig. 4B, respectively.

**TABLE 1:** Performance of *L. donovani*–Aurora Kinase in the Serodiagnosis of Indian and Brazilian Visceral Leishmaniasis cases^*a*^

		Serum samples, No./Total, 95% CI								
Country and test	Antigen used	VL	PKDL	NEHC	EHC	OD	Total control	VL follow-up	AUC value	Cut-off value
		(Sensitivity, %)		(Specificity, %)				(Reactivity, %)		
INDIA										
ELISA	LdAIRK	98.73 (78/79) 93.17–99.94	…^*b*^	100 (25/25) 86.68–100	100 (14/14) 78.47–100	79 (11/14) 52.41–92.43	94.34 (50/53) 84.63–98.46	8.3 (2/24) …	0.987	0.3
		**…**	93.75 (15/16) 71.67–99.68	94.74 (18/19) 75.36–99.73	…	…	94.74 (18/19) 75.36–99.73	…	0.983	0.24
DIPSTICK	LdAIRK	100 (30/30) 88.43–100	83.3 (10/12) 51.59–97.91	100 (20/20) 83.16–100	100 (10/10) 69.15–100	100 (10/10) 69.15–100	100 (40/40) 91.19–100	10 (1/10) …	…	…
LFT	LdAIRK	100 (25/25)	100 (15/15)	90 (18/20)	90 (9/10)	80 (08/10)	87.5 (35/4^*a*^0)	20 (3/15) …	…	…
	rK39	100 (25/25)	80 (12/15)	100 (20/20)	100 (10/10)	100 (10/10)	100 (40/40)	86.66 (13/15) **…**	…	…
BRAZIL										
ELISA	LdAIRK	97.5 (39/40) 86.84–99.94	…	84.21 (16/19) 60.42–96.62	…	…	84.21 (16/19) 60.42–96.62	…	0.978	0.45
DIPSTICK	LdAIRK	100 (27/27) 87.23–100	…	84.21 (16/19) 60.42–96.62	…	…	84.21 (16/19) 60.42–96.62	…	…	…
LFT	LdAIRK	100 (20/20)	…	84.21 (16/19)	…	…	84.21 (16/19)	…	…	…
	rK39	65 (13/20)	…	100 (19/19)	…	…	100 (19/19)	…	…	…

^*a*^ Abbreviations used in the study include EHC, endemic healthy controls; NEHC, non-endemic healthy controls; OD, other diseases; FU, follow-up patients; AUC, area under the curve.

^*b*^ “...” signifies that there is no data.

### ELISA test with Ldairk for reactivity with follow-up samples

To assess treatment response, we analyzed the presence of antigen-specific antibodies against Ldairk in follow-up (FU) serum samples. A total of 24 FU specimens were collected from Indian patients 6 months after treatment with AmBisome for *Leishmania* infection. ELISA analysis revealed a significant decrease in antibody reactivity, with only 8.3% of FU samples testing positive (Fig. 3A), compared with pre-treatment levels. A few FU samples exceeded the cutoff of 0.29, indicating minimal reactivity. For a more detailed analysis, paired serum samples from seven *Leishmania*-infected patients were tested at three time points: pre-treatment, 1 month post-treatment, and 6 months post-treatment. Antibody levels against LdAIRK sharply declined after 30 days, reaching very low levels by 180 days post-treatment (Fig. 3E). These findings indicate that the Ldairk antibody ELISA assay is promising for monitoring treatment response, but further longitudinal studies are needed to fully assess its prognostic potential.

### Dipstick assay of Ldairk with Indian and Brazilian serum

**Fig 5:**
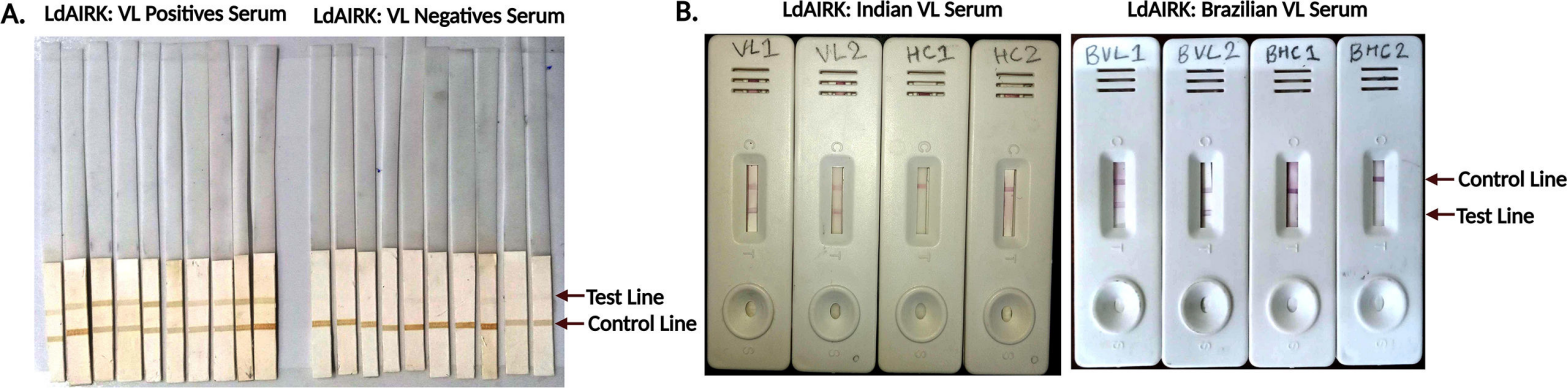
Schematic of dipstick immunochromatographic (**A**) and lateral flow assay (LFT) (**B**) using recombinant Ldairk. Both assays show a positive result with two bands (test and control) and a negative result with a single control band. LFT was validated using serum samples from Indian VL patients (VL) and healthy controls (HC), as well as Brazilian VL patients (BVL) and healthy controls (BHC).

Following the confirmation of Ldairk’s immunogenicity via immunoblot and ELISA, we evaluated its diagnostic potential in field settings using a dipstick assay, reducing diagnosis time from 8 to 2 hours. The assay tested 30 Indian VL serum samples, 10 PKDL, 20 NEHC, 10 EHC, 10 OD, 27 Brazilian VL samples, and 19 healthy controls. Dipstick analysis showed excellent reactivity, with positive bands appearing at both the test and control lines in all Indian and Brazilian VL serum samples, yielding a sensitivity of 100%. (95% CI: 88–100). For the healthy controls from India, including NEHC, EHC, and OD, all bands appeared at the control region with a specificity of 100% (95% CI: 91.19–100). In contrast, for Brazilian samples, there were three cross-reactive bands in the test line, resulting in a specificity of 84.21% (95% CI: 60.42–96.62). Additionally, with Indian PKDL samples, the sensitivity was 83.3% (95% CI: 51.59–97.91). Furthermore, dipsticks with follow-up (FU) samples from 10 Indian patients post-6-month treatment revealed 10% reactivity to Ldairk antigen, indicating the assay’s potential for distinguishing active VL cases from past infections (Fig. 5A, Table 1).

### Performance of lateral flow test (LFT) with human serum samples

**Fig 6:**
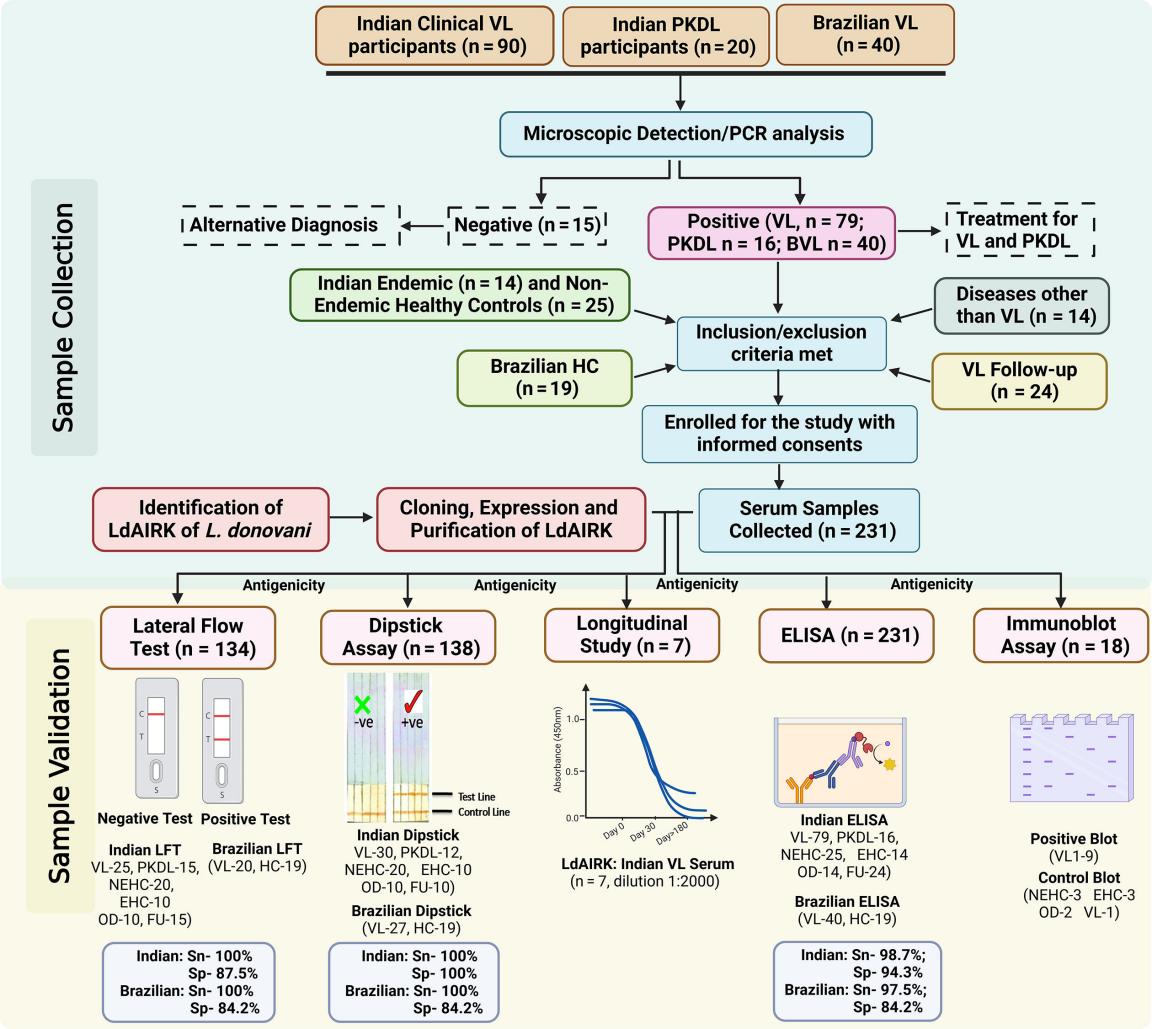
Proposed diagnostic flowchart for visceral leishmaniasis patients and healthy control serum samples from endemic regions of India and Brazil and its validation with different assays. Inclusion criteria: participants aged 18–60 years must present with fever (>99°F) and at least one of the following: weight loss, decreased appetite, enlarged spleen, or anemia. They should be clinically stable and willing to attend outpatient follow-ups. Additionally, patients with diseases other than kala-azar or stable healthy controls are eligible, provided they give written informed consent, or their parent/guardian does so if under 18 years old. Exclusion criteria: pregnant women, individuals who are HIV or COVID positive and co-infected, as well as those with limited capacity to consent or belonging to vulnerable social groups. Abbreviations: VL, visceral leishmaniasis; EHC, endemic healthy controls; NEHC, non-endemic healthy controls; OD, other diseases; FU, follow-up patients; Sn, sensitivity; Sp, specificity.

Building on the success of the dipstick assay, we developed a lateral flow test (LFT) for Ldairk, offering faster and more convenient results. Serum samples from VL patients in India and Brazil, along with non-VL controls, were tested and compared with the commercially available rK39 strip test. Among 25 *L*. *donovani*-infected VL patients from India, all VL-positive samples exhibited distinct test and control bands with the Ldairk LFT, indicating 100% sensitivity, which aligned with the results of rK39. For 15 cases of post-kala-azar dermal leishmaniasis (PKDL) tested, Ldairk demonstrated absolute reactivity with a sensitivity of 100%, whereas rK39 showed three false negatives, resulting in a sensitivity of 80%. The specificity of the Ldairk test was evaluated using 40 non-VL samples, which included 20 non-endemic healthy controls (NEHC), 10 endemic healthy controls (EHC), and 10 samples from patients with other diseases (OD). Among these, five samples (two NEHC, one EHC, and two OD, including cases of malaria and typhoid) yielded false positives, leading to an overall specificity of 87.5% for the LFA. Interestingly, follow-up samples taken 6 months after VL treatment showed 20% reactivity with the Ldairk LFT, whereas 86.66% of these samples remained positive with the rK39 test. This suggests strong diagnostic potential of Ldairk in India, especially to differentiate between recent and past VL infection. Similarly, a comparative study of the Ldairk-based LFA and the rK39 test was conducted using Brazilian serum samples, including 20 confirmed cases of visceral leishmaniasis (VL) and 19 healthy controls. The Ldairk LFA exhibited a sensitivity of 100% for Brazilian VL samples, whereas the rK39 test showed a lower sensitivity of 65%. Regarding specificity, the Ldairk demonstrated 84.21% with the healthy controls, whereas the rK39 test achieved a specificity of 100%. A prototype representative of Ldairk dipstick and LFT is illustrated in Fig. 5A and B, with the summarized results presented in Table 1. Fig. 6 illustrates a schematic overview of the antigenicity of LdAIRK using serum samples from visceral leishmaniasis patients and healthy controls from endemic regions in India and Brazil.

## DISCUSSION

Early, accurate, and affordable diagnosis is critical for the effective treatment and control of *Leishmania* transmission. Rapid diagnostic tests (RDTs) are cost-effective, adaptable to field settings, and provide quick results. However, commercially available RDTs, like rK39, show varying sensitivity and specificity across endemic regions, performing well in India but less so in Africa and Brazil (16). Additionally, they are unsuitable for post-treatment monitoring (17). Thus, there is a need for new antigenic candidates to improve the accuracy of VL diagnosis and confirm cure. This study aimed to address this gap by evaluating the serological performance of Aurora kinase LdAIRK, a highly conserved kinase protein, in Indian and Brazilian VL serum samples, as well as in serum from Indian post-kala-azar dermal leishmaniasis (PKDL) patients. LdAIRK was cloned, purified, and assessed using immunological assays, including ELISA, dipstick, and lateral flow assays (LFA). The antigen showed strong, specific recognition of human anti*-L*. *donovani* IgG antibodies in Indian samples and anti*-L*. *infantum* IgG antibodies in Brazilian samples. Bioinformatics analysis through NCBI BLAST revealed that LdAIRK shares 99.67%–100% identity with LiAIRK from *L. infantum* and other African strains. This makes Ldairk a promising candidate for developing diagnostic tests for both *L. donovani* and *L. infantum* infections in diverse endemic regions globally.

Kinesin-based antigens, including rK28, rK26, rKRP42, rKE16, rKLO8, and rKLi8.3, have been developed for ELISA, dipstick, and RDT formats to detect antibodies in patients with VL across endemic regions (18–21). Commercially available kinesin antigens rK39 and rK28 have shown variable performance in VL diagnostics globally (22) (23). Although rK39 demonstrates 97% sensitivity in the Indian subcontinent, it drops to 85% in East Africa, with intermediate results in Brazil (88%–94%) (16). rK28 shows higher sensitivity (99%, 96%, and 98% in India, East Africa, and Brazil, respectively), but with lower specificity in East Africa (83%) (24). A 2023 study by Mahdav et al. reported improved diagnostic efficiency with rKLi8.3 over rK39 in East Africa, although further validation with negative controls is needed (21). These findings highlight the necessity for new recombinant antigens beyond kinesins to address performance variability in diverse endemic regions.

Our study with the kinase-based recombinant antigen Ldairk began with membrane-based immunoblot assays to assess its reactivity with VL-specific IgG antibodies. Initial testing on 18 serum samples from India showed strong reactivity with VL samples and mild reactivity with controls. This pilot phase aimed to confirm the antigen’s antigenicity. Encouraged by these results, we expanded the study to include 231 samples for a more comprehensive ELISA analysis, transitioning from a validation to a broader diagnostic assessment. The Ldairk ELISA demonstrated excellent sensitivity (>97%) and an area under the curve (AUC) greater than 0.97, indicating strong diagnostic performance. This high efficacy was consistent across both Indian and Brazilian VL samples. However, some cross-reactivity in Brazilian negative controls led to slightly lower specificity compared with India. Additionally, Ldairk effectively detected antibodies in PKDL patient sera, further demonstrating its potential as a reliable tool for diagnosing VL and monitoring treatment response. Diagnosing PKDL is vital due to its potential to harbor *Leishmania* parasites in the skin, creating an additional reservoir for infection. Despite its non-fatal nature, PKDL frequently eludes detection in VL surveillance initiatives (25). Analysis of ELISA data with PKDL sera revealed exceptional antigen-antibody titers with 93.75% sensitivity and an AUC value of 0.983.

Transitioning from ELISA to dipstick assays represents a leap toward field-applicable diagnostics for VL. We evaluated the efficacy of Ldairk on dipsticks with VL, PKDL, and healthy control samples. Ldairk exhibited clear positivity with VL serum samples with 100% sensitivity and no reaction with negative controls, which include endemic, non-endemic healthy controls, and other diseases. However, false negativity with two PKDL serum samples occurred, demonstrating 83.3% sensitivity. Additionally, achieving 100% specificity with negative controls of serum indicates that the Ldairk-based dipstick assay has a better affinity with samples compared with ELISA.

The serum dipstick results were corroborated by LFT, which is more user-friendly, time-saving, and adaptable to field settings. The Ldairk demonstrated 100% sensitivity for detecting VL and PKDL using serum samples over the rK39 test, which retained a sensitivity of 80% with the same number of PKDL samples. Although there were instances of cross-reactivity with non-endemic and endemic healthy controls, and other diseases like malaria, and viral fever samples in lateral flow assays, overall, Ldairk-LFT shows promise for rapid and accurate diagnosis of Indian VL and PKDL samples.

Using Brazilian serum samples, both the Ldairk-based dipstick and LFT showed clear positivity in VL samples, achieving 100% sensitivity. This indicates that Ldairk has a significant advantage over the rK39 test, which demonstrated a lower sensitivity of 65% for detecting Brazilian VL cases. However, three instances of cross-reactivity with healthy controls were observed in both the Ldairk-dipstick and LFT formats, resulting in a specificity of 84.21%, compared with the rK39 test, which exhibited a clear specificity of 100%. Given the comparable outcomes with the aforementioned assays based on rK39 and rK28 (26), our Ldairk-based ELISA, dipstick, and RDT present potential for diagnosing VL caused by *L. infantum*. However, further investigations utilizing a larger sample size are necessary.

In regions where VL is prevalent, antibodies against the parasite can persist for many years following an episode of the disease. This persistence may result from repeated exposure to *L. donovani* or incomplete eradication of the parasite. In a longitudinal study, we observed a significant decrease in Ldairk-specific antibody levels in serum samples after 180 days of treatment. In ELISA, continued positivity in two serum samples signaled potential future relapse requiring continued disease monitoring. The findings from ELISA were supported by dipstick and lateral flow tests, indicating a notable reduction in antibody reactivity to the Ldairk antigen 6 months post-treatment. Ldairk exhibited a distinct advantage over the rK39 test, which retained 86.66% reactivity even after 6 months of treatment. To further assess this decline in reactivity with follow-up samples, measuring immunoglobulin G1 (IgG1) against Ldairk could be particularly insightful. A recent study highlighted that IgG1 detection against rK39 improves the monitoring of treatment outcomes in VL, as IgG1 levels may decline rapidly without a consistent and appropriate antigenic stimulus (27) (28). Our findings highlight the prognostic and diagnostic potential of serum-based ELISA and dipstick assays, warranting further investigation with samples from other endemic regions.

Although our findings are promising, the study has some limitations. We relied on clinically confirmed VL cases, which restricted our ability to predict VL positivity in participants. Additionally, we excluded samples from patients with other VL-related conditions or those co-infected with HIV. The study also lacked certain control groups in Brazil, and future validation studies in Brazil will address these gaps, focusing on diseases like cutaneous leishmaniasis and other neglected tropical diseases. Furthermore, the test’s performance is highly dependent on the cutoff value, which affects sensitivity and specificity. Despite these constraints, the Ldairk antigen demonstrated promising results for rapid VL diagnosis and treatment monitoring. These findings warrant further investigation in subsequent phases, including larger, more diverse samples from different endemic regions, particularly East Africa. Developing effective diagnostic tools for VL is essential for controlling this neglected tropical disease and advancing the United Nations' Sustainable Development Goal of Universal Health Coverage (29).

## MATERIALS AND METHODS

### Biological samples

For this study, we utilized a total of 231 archived human sera (Fig. 6). Of these, 147 samples were collected from the School of Tropical Medicine (STM) in Kolkata and the Rajendra Memorial Research Institute of Medical Sciences (RMRIMS) in Patna, India. An additional 25 samples were gathered from healthy volunteers at the CSIR-Indian Institute of Chemical Biology. Furthermore, we included 40 serum samples from visceral leishmaniasis (VL) patients caused by *L. infantum* and 19 control samples obtained from the Universidade Federal do Piaui in Teresina, Brazil. These Brazilian samples were transported to India in a frozen state and stored at −80°C until analysis. We excluded individuals who were pregnant, HIV or COVID-19 positive with co-infection, had limited capacity to provide consent, or belonged to vulnerable social groups.

The VL cases initially screened through rK39 (Kalazar Detect Rapid Test, InBios International, Inc., USA) were confirmed parasitologically via microscopic examination, whereas post-kala-azar dermal leishmaniasis (PKDL) cases were confirmed through PCR of skin specimens. Among the Indian samples, there were 79 from patients with active VL (AVL), 16 from PKDL patients, 14 from close relatives of patients who served as endemic healthy controls (EHC), and 33 from non-endemic healthy controls (NEHC). Additionally, 14 samples were from patients with other diseases (OD) presenting symptoms similar to VL, including five from malaria, five from viral fever, and two each from typhoid and tuberculosis, collected from hospitals. Follow-up samples, totaling 24, were collected approximately 6 months post-treatment of VL patients. Samples from EHC, NEHC, and OD patients were used as negative controls, whereas samples from AVL and PKDL patients served as positive controls, and FU samples were used to assess reactivity with the antigen. All samples were stored at −20°C until further use.

### Cloning, overexpression, and purification of *L.d*AIRK

The process of cloning, overexpression, and purification of LdAIRK followed a previously outlined protocol (15). In brief, TritrypDB version 3.2 (http://tritrypdb.org/tritrypdb/) and TDRtargets (http://tdrtargets.org/) were used to select novel druggable targets in kinetoplastid parasites. Nucleotide and amino acid sequences of selected proteins were retrieved from the GeneDB gene database. Sequences from other model organisms were obtained from NCBI. Using BLAST through NCBI (http://blast.ncbi.nlm.nih.gov/Blast.cgi) and GeneDB (http://www.genedb.org), we focused on minimizing similarity to human proteins. The model organisms for these proteins are detailed in Chhajer et al., 2016 (15). We identified LdAIRK, recognized for its essential roles in cell division and virulence. The GenBank accession numbers for LdAIRK are XM_003862106 (nucleotide) and XP_003862154 (protein). Initially, the LdAIRK gene sourced from the *L. donovani* AG83 strain (ATCC PRA-413) was inserted into the pET28a vector utilizing NcoI/HindIII restriction sites and subsequently expressed within Rosetta (DE3) strain of *E. coli* cells. Overexpression of the Ldairk protein was conducted at a temperature of 37°C with 0.3 mM isopropyl-β-D-1-thiogalactopyranoside (IPTG) when the optical density (OD) reached approximately 0.4–0.6. Following a 4-h induction period, the cultures were harvested, and the cell pellets were suspended in bacterial lysis buffer. This buffer contained 25 mM Tris-HCl, 300 mM NaCl, 1 mg/mL of lysozyme (Roche), and 1 mM phenylmethylsulfonyl fluoride (PMSF) at a pH of 8.0. Sonication was then employed using an ultrasonicator (Misonix, Farmingdale, NY, USA), followed by centrifugation of the lysates for 30 min at 12,000 rpm to collect inclusion bodies. Subsequently, the inclusion bodies were solubilized in a binding buffer composed of 6M urea, 10 mM imidazole in 25 mM Tris-buffered saline (TBS) and then centrifuged at 11,000 rpm for 30 min to gather the supernatants. These supernatants were added to pre-equilibrated Ni-NTA agarose matrix to facilitate the binding of His-tagged proteins. The bound matrix underwent two washes with 50 mM imidazole-containing urea buffer with 0.1% Triton X100, followed by two additional washes without Triton X100. Finally, the bound recombinant Ldairk protein was eluted using an elution buffer containing 500 mM imidazole. The urea-denatured eluted fractions were dialyzed gradually to decrease the concentration of urea, ensuring proper folding of the proteins. Using Amicon Ultra centrifugal filter devices from Millipore Corporation, USA, the protein fractions were concentrated. Purification of the proteins was verified using a 10% SDS-PAGE gel, followed by staining with Coomassie brilliant blue. Protein concentrations were determined for each sample using Lowry’s method (30).

### Immunoblot assay

Immunoblot assay/western blot against recombinant Ldairk antigen was performed on 10% SDS-PAGE. The resolved proteins were transferred electrophoretically onto a 0.45 µm nitrocellulose membrane using a transblot apparatus from Bio-Rad Laboratories, USA, at a constant current of 1.5 A for 15 min. After confirming the protein transfer with Ponceau S staining, each lane was cut into strips and blocked with 5% BSA in TBS for 60 min. The strips were then incubated overnight at 4°C with constant shaking, using primary antibodies from serum at a 1:2,000 dilution. The following day, HRP-conjugated goat anti-human IgG secondary antibodies diluted at 1:3,000 were added and incubated at room temperature for 1 h with constant shaking. Each strip was washed five times with wash buffer at 5-min intervals, followed by the addition of chemiluminescent Horseradish Peroxidase (HRP) substrate (Sigma-Aldrich, USA) to all the strips (blot). Using the Gel Doc system from Bio-Rad, images were developed within 20 s and analyzed with Image Lab software (version 54.2.1, Bio-Rad) (31).

### Enzyme-linked immunosorbent assay

To evaluate the performance of recombinant Ldairk, an enzyme-linked immunosorbent assay was conducted to measure IgG antibodies specific to Ldairk antigen. Recombinant Ldairk antigen (1 mg/mL) was coated onto 96-well ELISA plates (Nunc) at 100 µL per well and incubated overnight at 4°C in 10 mL phosphate buffer (PB). The following day, after washing twice with phosphate-buffered saline with 0.05% Tween 20 (PBST), the wells were blocked with 1% bovine serum albumin (BSA) in PBS buffer (200 µL/well) at 37°C for 1 h. After three washes with PBST, serum samples (diluted 1:2,000) (11) containing primary antibodies were added at 100 µL per well and incubated at 37°C for 2 h. Subsequently, secondary antibodies (HRP-conjugated anti-human IgG, diluted 1:3,000 in PBS buffer) were added and incubated at 37 °C for another 2 h. Following five washes with PBST, bound IgG was detected within 2 min using 3,3',5,5'-tetramethylbenzidine (TMB) substrate (Sigma-Aldrich). The reaction was stopped at room temperature by adding 50 µL 2N H_2_SO_4_, per well, and optical density values were measured at 450 nm using a microplate spectrophotometer (Thermo Fisher Scientific, USA).

### Dipstick preparation and assay

To create nitrocellulose membrane-based dipsticks suitable for field use, a membrane with dimensions of 2.4 × 8 cm and a pore size of 0.45 µm (GE Healthcare Life Sciences) was soaked in 25 mM Tris-HCl buffer (pH 7.6). When semi-dried, with a dispense rate of 5 µL/cm, 1 μg of Ldairk was dispensed onto the membrane at the test line, and a 1:10 dilution of rabbit anti-human IgG (unlabeled, Southern Biotech, USA) was dispensed at the control line as positive control using a Flowline F100 dispenser (Precore Solutions, Kochi, India). The membranes were incubated for 30 min at room temperature and then blocked overnight at 4°C with a solution containing 2% BSA, 0.1% Tween-20, and 0.01% NaN3 in 100 mM Tris Buffer Saline (TBS), followed by washing with TBS containing 0.05% Tween-20 (TBST). The next day, the membranes were dried and then affixed to a plastic sheet and cut into 0.4 mm diameter strips. These ready-touse dipstick kits were stored at room temperature and can be used for at least one year with simple desiccation until the experiment is performed. For testing, dipsticks were incubated in serum samples (diluted 1:2,000 in TBS). After incubation, the dipsticks were treated with enzyme-conjugated anti-human HRP-IgG (Southern Biotech, diluted 1:2,000). Subsequently, the strips underwent two thorough washes in TBST and one in TBS. The reaction was visualized by dipping the strips into a substrate solution composed of 0.05% 3,3'-diaminobenzidine tetrahydrochloride (DAB, Sigma, USA) containing 0.05% H2O2 in 100 mM TBS. The reaction was halted by dipping the strips in distilled water. Dark brown bands appearing at both the test and control lines within 3 min of the strip drying indicated a positive result for VL, whereas a single band at the control line indicated a negative result for VL (32).

### Lateral flow prototype development and assay

To prepare the lateral flow prototype, a 10 µm nitrocellulose membrane from mdi Membrane Technologies, India was coated with Ldairk (2 mg/mL) at the test line and protein G (2 mg/mL) at the control line as positive control using a reagent dispenser from Precore Solutions, India. The coated membrane was dried at 37°C for 30 min. A mixture consisting of colloidal-gold conjugated protein G (prepared with a 1:2 ratio with 10% sucrose in PBS buffer) from Ubio Biotechnology Systems, India was then coated onto a conjugate membrane and similarly dried at 37°C for 30 min (25). Subsequently, the sample membrane, protein G conjugate membrane, test and control line coated nitrocellulose membrane, and absorbent membrane were assembled together and cut into 2.5 mm strips to be placed into a diagnostic plastic cassette until use. During the assay, a human serum of 10 µL was applied at the cassette orifice near the sample membrane position, followed by two times with 23 µL chase buffer containing 20 mM Tris (pH 7.4 with 0.1% Tween-20). After 5 minutes, the capillary action of the chase buffer and serum sample mixture allowed the visualization of pinkish-colored bands on the nitrocellulose membranes. If the control line was absent, the test was deemed invalid.

The “Kalazar Detect Rapid Test” (Kalazar Detect RDT) from Inbios International in Seattle, USA, was conducted according to the manufacturer’s guidelines using serum samples. During the assay, 10 µL of human serum was placed onto the device with a micropipette, followed by the application of chase buffer using the provided dropper. All samples were tested, and the results were recorded and compared with those from the Ldairk-RDT.

### Data analysis

GraphPad Prism 8.0 software was utilized for statistical analysis. Sensitivity and specificity of Ldairk ELISA cutoff values were determined using receiver operating curve (ROC) analysis with 95% confidence intervals. The parameters of the ROC curve include the overall accuracy of the test, which is indicated by the area under the curve (AUC). This is a widely recognized metric for assessing diagnostic accuracy. A higher AUC indicates better diagnostic performance, with an AUC of 1 signifying perfect accuracy. Additionally, statistical significance was assessed using the Mann-Whitney U test, with differences considered significant at a *P* value < 0.05.

## Data Availability

All the information provided in the research is contained within the article. For additional information, please contact the corresponding author.
